# Systemic bacterial infection induces cerebrovascular dysfunction and exaggerated neuroinflammation in APP/PS1 mice

**DOI:** 10.1002/alz70855_105435

**Published:** 2025-12-24

**Authors:** Matthew P Colley, Aaron B Kantor, Jessica Teeling

**Affiliations:** ^1^ University of Southampton, Southampton, Hampshire, United Kingdom; ^2^ Therini Bio, Sacramento, CA, USA

## Abstract

**Background:**

Alzheimer's Disease (AD) is characterised by the accumulation of aggregated β‐amyloid and neurofibrillary tangles, with the immune system being a key driver of AD pathology and progression. Systemic infections can worsen cognitive decline in patients, possibly due to cerebrovascular dysfunction and exaggerated neuroinflammation, but the underlying mechanisms are not fully understood. Modelling systemic infection in animal models has classically been carried out using lipopolysaccharide (LPS) to mimic a systemic bacterial infection. LPS activates the innate immune compartment of the immune system but does not accurately represent the chronic low grade inflammatory changes observed during a real bacterial infection. In this study, we investigate the inflammatory and vascular effects following exposure to a live, attenuated bacterial strain *Salmonella Typhimurium* in APP/PS1 and WT mice.

**Method:**

APP/PS1 mice and WT littermates were infected with 1x10^6^ CFU *Salmonella Typhimurium* (SL3261) or sterile saline control by intraperitoneal injection and tissue from perfused mice was collected at 7 and 28 days post‐infection. Liver and brain tissue were analysed for systemic inflammation, cerebrovascular dysfunction and neuroinflammation using immunofluorescence.

**Result:**

We report that systemic infection with *Salmonella Typhimurium* induces fibrin(ogen) deposition in the liver accompanied by the formation of FcγRI and CD11b positive inflammatory lesions. Cerebrovascular activation, measured by increased expression levels of VCAM and ICAM, is observed 7 days post infection, which is accompanied by extravasation of fibrin(ogen) and IgG into the brain parenchyma. Microglial activation, measured by expression of MHC‐II and FcγRI, revealed an exaggerated microglial response in APP/PS1 mice when compared to WT littermates. The cerebrovascular dysfunction following exposure to *Salmonella Typhimurium* may have contributed to this exaggerated neuroinflammatory response.

**Conclusion:**

This study shows that APP/PS1 mice with a systemic bacterial infection have exaggerated microglial neuroinflammatory response. The cerebrovascular dysfunction and fibrin‐ mediated activation of microglia following exposure to *Salmonella Typhimurium* may have contributed to this exaggerated neuroinflammatory response.

